# Experimental Study of Abrasive, Mechanical and Corrosion Effects in Ring-on-Ring Sliding Contact

**DOI:** 10.3390/ma13214950

**Published:** 2020-11-04

**Authors:** Jaroslaw Selech, Dariusz Ulbrich, Dawid Romek, Jakub Kowalczyk, Konrad Wlodarczyk, Karol Nadolny

**Affiliations:** Department of Civil and Transport Engineering, Institute of Machines and Motor Vehicles, Poznan University of Technology, 60-965 Poznan, Poland; jaroslaw.selech@put.poznan.pl (J.S.); dawid.romek@put.poznan.pl (D.R.); jakub.kowalczyk@put.poznan.pl (J.K.); konrad.wlodarczyk@put.poznan.pl (K.W.); karol.nadolny@put.poznan.pl (K.N.)

**Keywords:** abrasive wear, corrosion wear, heat treatment, ring-on-ring test, sliding contact

## Abstract

This article presents the application of the ring-on-ring test to investigate some of the important factors affecting the abrasive and corrosion wear of a face seal used in the sugar industry. The test involves the sliding contact between two steel rings working in different conditions such as mechanic, abrasive, corrosive extortions and its combination. Rings were made of the C45 steel and the surface layers were modified by heat and thermochemical treatment such as normalizing, flame hardening, nitriding and chrome diffusion. Maximum wear of the sample after tests under mechanic, abrasive and corrosion extortion were obtained. For C45 steel without surface modification the biggest wear was obtained for mechanical, abrasive and corrosive extortion and equals 0.0138 g. This value was three times bigger than the result for the mechanical extortion and ten times than for the corrosive conditions. For individual research options the percentage increase or decrease in wear resistance in relation to the normalized surface layer was determined. In the corrosive extortion the highest increase (90%) of wear resistance was recorded for the chrome layer relative to normalizing sample. The main conclusion of the paper is that the wear effect caused by all factors—mechanical, abrasive and corrosive—is not a straight sum of values of wear.

## 1. Introduction

Elements of machinery and equipment in the food industry, in many instances, work in technologically forced aggressive environments. Therefore, materials designed specifically to work as operating elements of the machinery and equipment in that industry must be characterized not only by appropriate strength and resistance against abrasive wear but also have specific characteristics of the surface layers, which are crucial in corrosion resistance [1]. Many components made of different materials must be resistant to both abrasive and corrosive wear [2]. Corrosion processes, which occur together with natural tribological enforcements, in many cases, substantially change the mechanisms of tribological phenomena. It is reflected in reduction of durability of friction nodes and in particular, working elements exposed to aggressive substances. Wear of machine and equipment elements caused by two components at the same time: abrasive wear and corrosion, is not a sum of two values resulting from two separate processes but depends on a number of factors [3,4,5].

There is a factor that itself largely decides about the intensity of process of simultaneous abrasive and corrosive wear, namely the initial state of surface layer of mating working surfaces. If there is consciously control of the characteristics of the surface layer it will be able to learn about initial effects of such phenomena by observing the wear effects in modeled abrasive and corrosive wear conditions. This type of research makes it possible to define influences of individual parameters characteristic of the surface layer, on the process of wear in working surfaces of kinematic nodes and, simultaneously, to define the effects, that is magnitude of the tribological wear.

Research on material wear in various conditions is conducted by many scientists around the world. Wieczorek [6] performed on ring-on-ring test stand research and determined the volume loss of the sample. The main conclusion of the research is that the wear resistance of hard-wearing steels was approximately four times higher as compared to S355J2 structural steel. The same test was used by Zanoria [7] for abrasive wear modeling of rolling undercarriage components in track-type machines. Block on ring sliding wear evaluation in different environments such as normal loading, speed, corrosion and lubrication includes adhesion wear, two-body abrasion, three-body abrasion and fatigue wear [8]. The same test was used to weight loss evaluation of the sample consist of the Cr-Mn-Si steel and the thermos analysis during the test was conducted [9]. Steel of the different microstructure but the same hardness was evaluated by Zambrano et al. [10] in block on ring test and the calculated wear coefficient showed a pronounced dependence with the yield strength of the steel.

In some conditions the tribocorrosion phenomena occurs (wear and corrosion at the same time). Cao et al. [11] presented different categories of the tribocorrosion model based on numerical methods for estimating time transition in tribocorrosion. A separate group consists of studies on the phenomenon of tribocorrosion in the sea water [12,13,14]. The articles contain the influence of individual factors on the wear process of various materials.

From the point of view of machine components and the environment in which they operate the method of preparing the surfaces of the cooperating elements in the node are important. Pawlus et al. [15] presented the method that allows obtaining the correct oil capacity in combination of the surface of elements. Various surface treatments are also used to create the desired properties of the cooperating surface layers [16,17,18]. Tests are also conducted in the scope of assessing the wear of elements in the abrasive mass [19]. Chosen research in the field of steel wear evaluation is presented in Table 1.

On the basis of the above list, it can be concluded that tests of various materials, including steel, with the use of various devices and environments were conducted. Senatorski et al. [21] presented the results of wear of the C45 steel in the corrosion environment. The sum of the abrasive increment in the wear test in a mixer stand with the use of fused alumina is presented in [22]. The methodology proposed by [3] allowed one to quantify the wear-corrosion synergism in aqueous tribosystems and it was specified that the effect of corrosion on total wear ranges from 0.1 to 4%, whereas the wear-corrosion synergism varied from ll to 92% of the total degradation. Diomidis et al. [33] described research about the tribocorrosion process of stainless steel in sulfuric acid. The material loss in the sliding track W_tr_ and other specific parameters were calculated. The three-body abrasion corrosion contact has been applied to determine whether tribo-electrochemical data can pick up any effects on the tribocorrosion characteristics [34]. Some of the research proved that wear rate of cast irons reach a maximum at around 35 percent sulphuric acid and increase with load and temperature [35].

Most of the tests described above concern wear tests other than the ring-on-ring test, which generates the need to perform tests in which the samples cooperate with the surfaces (frontal connection), as it occurs in the food industry. In addition, it seems important to determine the wear of the cooperating elements not only during the impact of the abrasive, but also mechanical extortion and the corrosive environment (sulfuric acid, which is used in the food industry), because all three of these factors occur in real conditions. So, the authors planned and determined the wear of cooperating samples for the connection occurring in the industry depending on the extortion (mechanical, abrasive and corrosive). This is a comprehensive approach, different from the publications described above, where usually one or two factors affect the wear results. A few of them like [34] were described. In addition to the base material, various types of surface layer treatments are proposed to check their wear resistance.

The aim of this research work was to determine the quantitative relationships between effects of the occurring destructive tribological processes and consciously varied initial technological states of surface elements in friction nodes. Such states were forced using special surface heat treatment and thermochemical treatment. Additionally, the relationship between the metallographic structure of the material after various treatments and wear of the sample was determined. The surfaces of the samples were also examined in order to determine its condition and the dominant factor degrading the surface layer. In addition, the friction moments for individual tests and changes in surface roughness parameters were determined.

## 2. Materials and Methods

### 2.1. Experimental Set-Up

**Figure 1:**
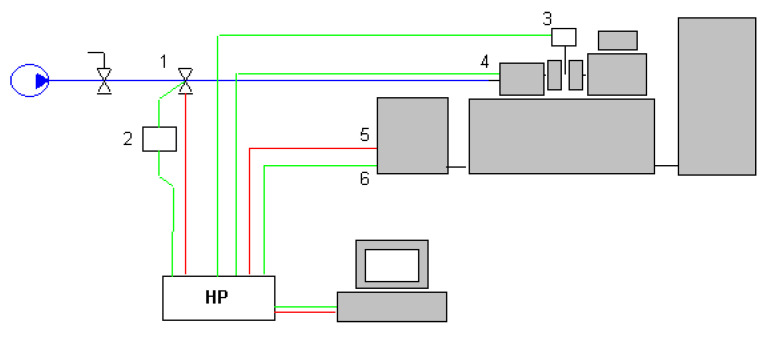
A control and measurement schematic of UMT-2168 friction testing machine [36]: 1—pneumatic valve, 2—4/20 mA armature, 3—thermocouple, 4—moment of friction converter, 5—0–1500 rpm generator control, 6—tachometric generator.

Tests were performed in the laboratory conditions, using UMT 2168, a specially modernized friction testing machine, made by Join Stock Company (Iwanowo, Russia). The machine is linked to a computer through a Hewlett-Packard 34970A multimeter (Palo Alto, CA, USA). A control and measurement schematic is shown in Figure 1.

**Figure 2:**
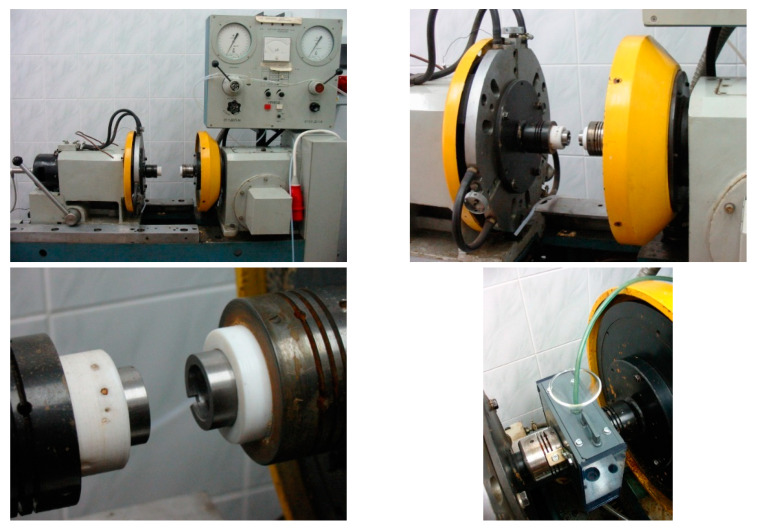
UMT 2169 friction wear machine.

**Figure 3:**
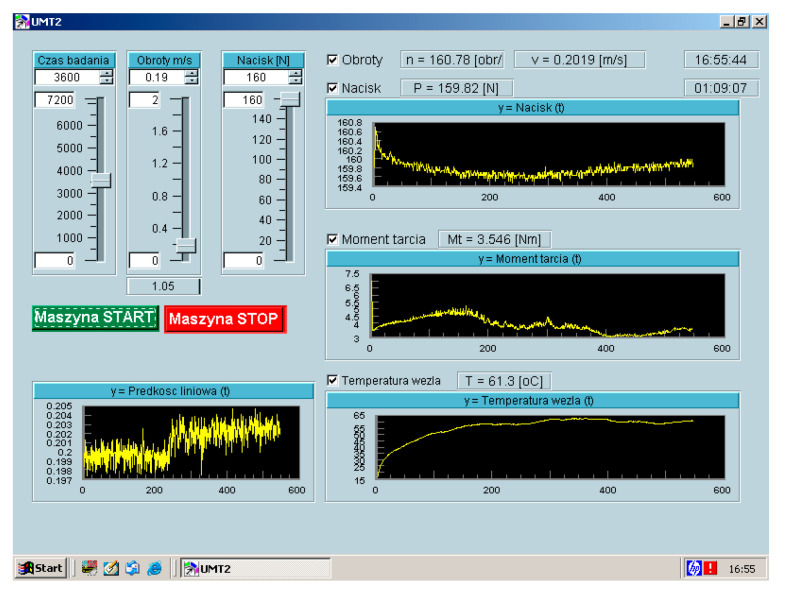
Machine control panel.

The system of force application and monitoring parameters is both controlled and displayed on the HP measurement device coupled with a computer. In order to program the software written in the HP VEE language (version 4.0) was used [37], which allowed for controlling all the experiment parameters such as friction torque, force applied to the sample and temperature. The measurement part consisted of the following: moment of friction converter, pressure exerting force applied to the sample converter, tachometric generator and a thermocouple. The friction wear machine is illustrated in the Figure 2 and the control panel in Figure 3.

**Figure 4:**
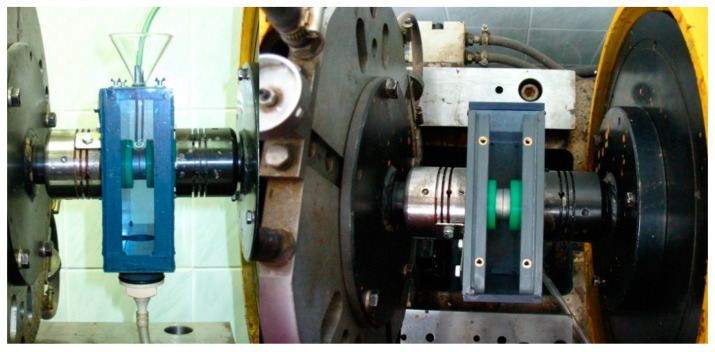
Testing chamber view.

In order to perform tests in the aggressive environment, the authors had to design and build a special testing chamber, including a system of supply and drainage of liquid stream and abrasive material. A view of the testing chamber is presented in Figure 4. It was built from plastic material, i.e., polyvinyl chloride (PVC) and Perspex (PMMA). Such a design made it easy to observe the working kinetic pair and to measure the temperature using a thermocouple [13].

**Figure 5:**
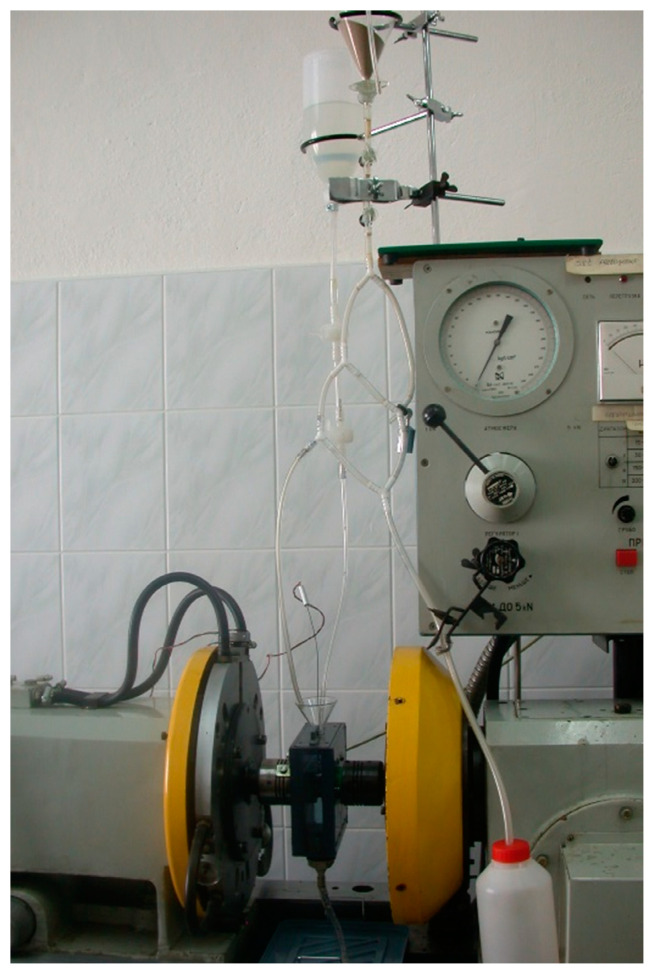
A view of the material supply system.

The system supplying liquid and abrasive material ensured stable conditions of node mating through constant inflow of corrosive liquid and abrasive material at a defined rate of quantity over time (Figure 5). It has been built from teflon hoses, flow valves, pear-shaped separators, three way pipes, a glass container, funnels, etc.).

### 2.2. Material Properties and Specimen Dimensions

As testing material, the authors chose the C45 steel. Its structure is ferritic-pearlitic, with a similar share of both phases. It is ordinary construction steel of higher quality and of general use. It is used both in its normalized state and in a thermally upgraded state and as surface hardened steel to make exposed to abrasion, medium load machinery parts, such as: wheels, gear shafts, rollers, shafts, axes, pins and circular cams, and some tools, such as: hammers, axes and wrenches and some plumbing fittings, and to make boiler or steam turbine equipment working in temperatures below 450 °C. The material is also often used in model tribological testing. It was delivered by a steel manufacturer, following the initial hot drawing treatment. Its chemical composition, basic mechanical and physical properties defined for the C45 steel are presented in Table 2 and Table 3.

Tests were performed using a ring–ring type kinematic node (sample and counter-sample), which mated frontally [38]. One of the rings (sample) was fixed, while the other (counter-sample) rotated at a defined sliding ratio (n) and force (N), changing with respect to the applied pressure.

**Figure 6:**
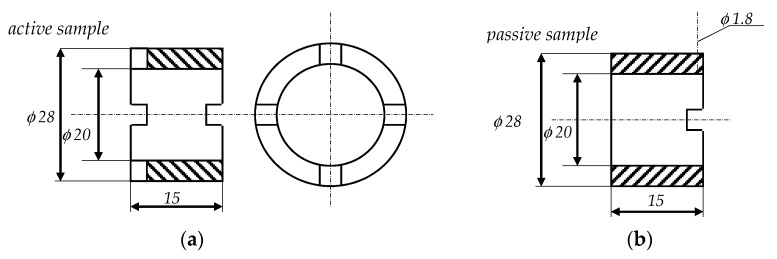
Sample dimensions: (**a**) active sample (the option with incisions) and (**b**) passive sample (the option without incisions) [40].

**Figure 7:**
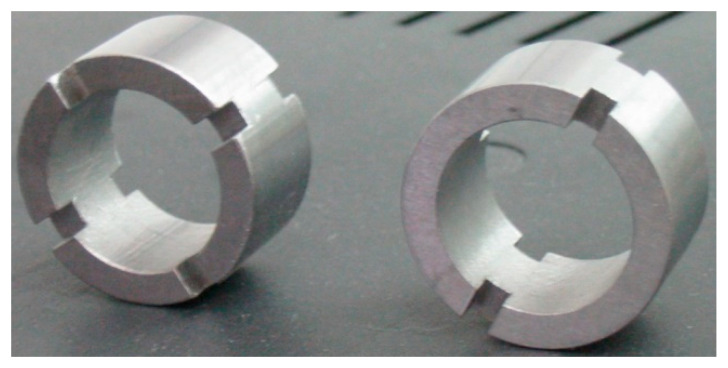
View of associated pairs.

In order to obtain an effect of speeding up the tests, the working surfaces of (active) samples, which were part of the node, were modified by cutting out the additional four incisions [39]. This resulted in supplying more abrasive material to the area of friction contact and intensified the process. Sample dimensions and view were shown in Figure 6 and Figure 7.

In order to ensure protection for surfaces not participating directly in the process of friction against corrosion, they were covered with a protective film made from bee wax. It had very good plasticity properties, did not react with the surface, covered it evenly with a thin film, which could be removed completely with ultrasounds and what is most important, protected the covered surface against the activity of the corrosive liquid. Test samples were given heat and thermochemical treatment, for details see Table 4. As a result of the treatments, different surface layer structures, and a differentiation of their characteristics was obtained. The resulting surface layers had different resistances to wear, depending on the type and intensity of the treatment.

All kinds of the selected heat treatment (normalizing, hardening with high tampering, hardening with low tampering) increasing the hardness and other mechanical properties should influence the increase of resistance to mechanical and abrasive wear. In the case of the thermochemical treatment (nitriding and chrome hardening), the properties of other diffusive values should influence the increase of resistance to corrosive wear.

**Figure 8:**
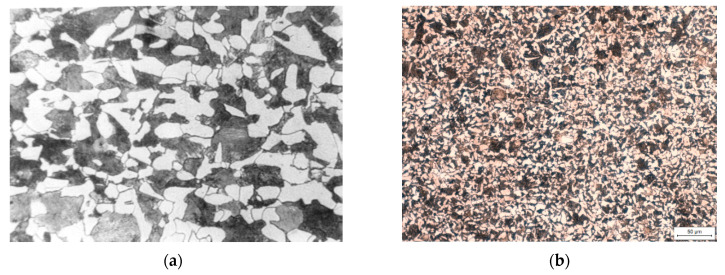

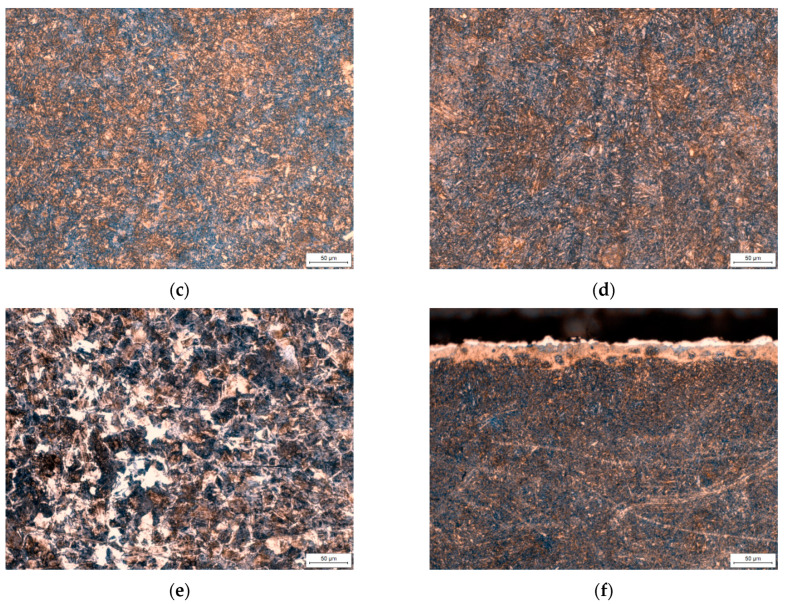
View of metallographic structures; (**a**) steel 45, (**b**) normalizing, (**c**) flame hardening + high tempering, (**d**) flame hardening + low tempering, (**e**) nitriding and (**f**) diffusion chrome hardening.

After the normalizing process (Figure 8b) a significant change in the grain size and uniformity was obtained compared to the C45 steel. Figure 8c shows highly tempered martensite composed of very fine spherical cementite particles in a ferritic matrix. In Figure 8d the structure had low-tempered martensite obtained as a result of flame hardening and low tempering, it is tetragonal martensite with dispersive ε-type carbides and residual austenite. In Figure 8e a structure after nitriding is presented. Thin zone of nitrides with a thickness of 2–3 µm and a martensitic-bainitic structure with a small amount of pearlite and ferrite were visible. Figure 4f shows the structure after chrome plating, which contained fine-grained martensite in the core, while in the surface layer 4 µm thick chromium was noticeable, repeated due to diffusion chrome plating.

### 2.3. Abrasive Characterization

**Figure 9:**
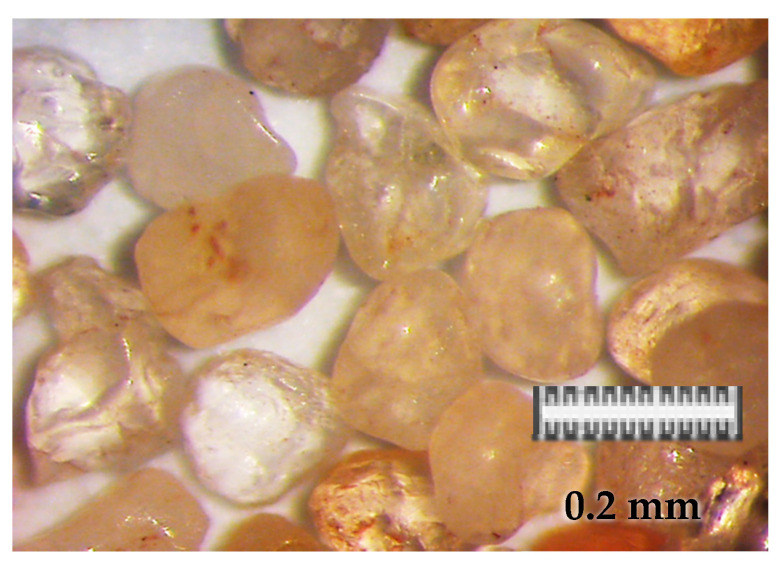
Shape and size of abrasive material.

The friction node was immersed in continuously supplied liquid, ensuring a constant temperature of node mating. Silicon sand was used as abrasive material, with a grain fraction at 0.2–0.3 mm, and hardness at 995% ± 10% HV, while the abrasive material supply rate was set at the level of 0.5 (g/cm^2^·s). The sand was selected in such a way that the shape of grains, degree of reeling, all matched the requirements of similarity to soil sands (Figure 9), in accordance with the PN-EN 933-1:2001 standard. The sand hardness was approximately 995% ± 10% HV. In order to get the right fraction and dispose of dust and organic pollutants, the sand was flushed, and a sieve analysis was performed, according to the PN-H-04188:1997 standard. The intensity of abrasive material inflow was set at the level of 0.5 (g/cm^2^·s) [1].

### 2.4. Test Parametres and Procedure

The temperature of the tribological process was controlled with a thermocouple, placed at the area of friction contact, and kept at a constant level (23 ± 3 °C) by regulating liquid inflow for all variants of the experiment. The duration of the experiment, for each kinematic pair, was 3600 s. Detailed data regarding conditions of mating for each node is included in Table 5.

Working surfaces of the samples were machined by polishing on a grinding wheel, therefore comparable parameters of roughness in all mating surfaces of samples and counter-samples were obtained.

The following parameters were registered in the course of tests: friction torque, pressure force, peripheral speed and temperature at the distance of 1 mm from the friction surface of the passive sample. Due to ongoing measurement of temperature in the course of the process, it was possible to stabilize it by changing the rate of flow of the coolant, which, in the case of this test, was a 10% solution of sulphuric acid, while distilled water was used for the purpose of abrasive and mechanical tests. Constant measurement of the moment of friction was used to estimate the quality of the course of the process. The stable moment of friction qualified such an implementation for further analysis. Ongoing control of quality of experimental processes (peripheral speed, temperature and moment of friction) made it possible to assume that the results are repeatable and, therefore, credible.

In order to remove all polluting agents from the samples, i.e., grease, sweat, dust, wear products, etc., all tested samples were cleaned in an ultrasound cleaning device and washed in a purpose made liquid, Eskapon E5060. Then samples were dried and kept in an exicator. The same procedure was applied before and after completion of wear tests. In order to properly assess errors, experiments were carried out in identical research conditions and were repeated at least five times.

The wear value measurement was performed using Sartorius model BP 221S analytical scales with an electronic display, which enabled measurements with reading precision of 0.0001 g, measurement error for this device was d = 0.1 mg.

Tests were performed for five options of heat treatment and thermochemical treatment. Input material was the C45 steel. The tests were divided into five stages, depending on the chosen tribological force application. In the first one, wear was only the result of mechanical wearing (with no abrasive material used), distilled water was both a lubricating and node heat conducting agent. In the second, abrasive material was introduced to the friction node, and the abrasive–mechanical wear effect was achieved. The third option focused on purely corrosive extortion, while the fourth, apart from corrosive, included also mechanical extortion. The final, fifth option was a simultaneous application of all three, i.e., mechanical, abrasive and corrosive extortions. The methodological options described above are presented in Table 6 [41].

It was assumed that nominally the pairs of options from the same category would be mated in each analyzed option of technological surface layer treatment.

## 3. Results and Discussion

### 3.1. Sample Properties

The hardness of the elements was tested using the Vickers method in accordance with PN-91-/H-04360. The method consisted of pressing a regular rectangular diamond cone into the tested material. HV01 to test the microhardness of the surface layer and HV1 to test hardness of the core were designated. The tests were carried out on 3 randomly selected samples, 15 measurement for each variant of the surface treatment were conducted. The test results are presented in Table 7 and Table 8.

**Figure 10:**
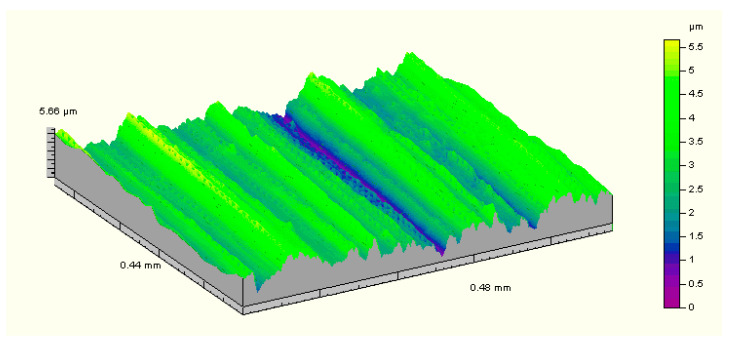
View of the geometric structure for flame hardening + high tempering.

The geometric structure of the surface had a very significant influence on the operational properties. It is characterized by shape errors, secondary texture, roughness and directivity. In tribological tests roughness is the most important factor. Other features such as secondary texture or structure defects can be eliminated by using methods ensuring high technological quality. The exemplary results of the 3D surface profile obtained on a Hommelwerke T8000 profilometer (VS-Schwenningen, Germany) using the M1-DIN-477 filter (VS-Schwenningen, Germany) are presented in Figure 10 and Table 9 presents the average results of geometric structure parameters.

Paper presented only the Rv parameter, which is the distance between the deepest valley of the profile, because from a physical point of view, the Rv correlation illustrates the fact that a surface with deep valleys (high value of Rv) induced a high stress concentration at the root of the surface profile, which could reduce the corrosion resistance. Besides that Rv parameter was not sensitive to process changes, relatively robust, great scratch identifier used for ground and polished surface.

**Figure 11:**
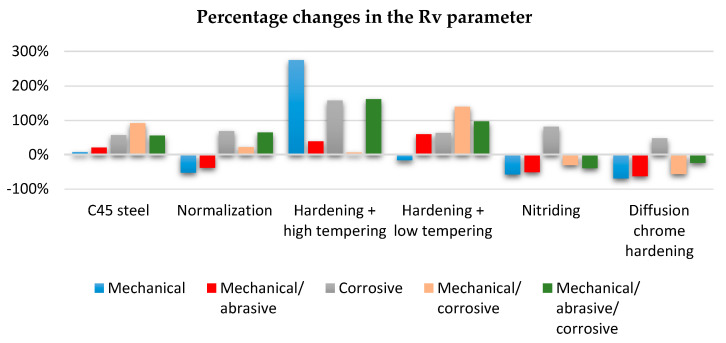
The change of the Rv parameter value obtained after friction tests to the value before the wear test.

Table 10 and Figure 11 shows the percentage changes in the values of the maximum roughness heights Rv that were reached after the tests compared to the values before the test. Positive values indicate an increase in the tested parameter and negative values indicate its decrease.

The following conclusions can be drawn from the obtained results:

(1) In the process of tribological wear, the thermochemically treated surface layers (nitriding and chroming) were smoothed, which indicates a decrease in the Rv parameter.
(2) After the corrosive wear processes, there was an increase in roughness expressed by the Rv parameter.
(3) As a result of wear tests, there was no clear tendency for the Rv parameter.

The results of 3D surface layers measurements of the geometrical structure of the surface, both before and after the wear test, revealed the specific features of these surfaces, which were not shown by standard 2D surface layers measurements. These were characteristic depressions and elevations, which the shape and direction did not coincide with the direction of movement of the rubbing surfaces. This gives a more complete knowledge of the influence of the tested surface geometrical structures on the operational properties and performance parameters of the tested friction nodes.

### 3.2. Friction Torque Calculation

**Figure 12:**
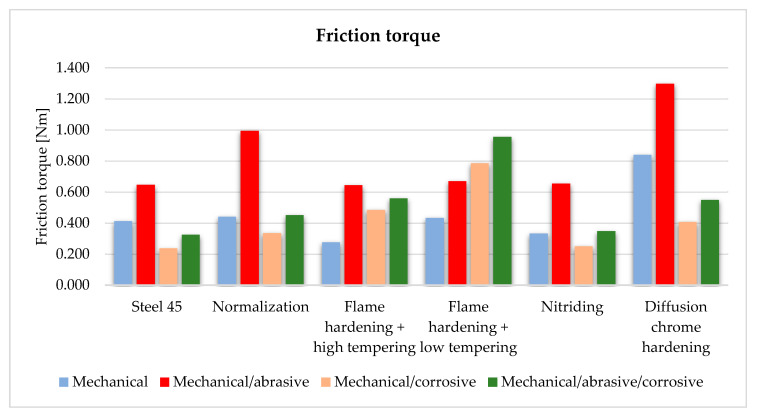
Friction torque for all the samples.

For the C45 steel the highest value of the friction torque was obtained for the 2nd variant of excitations (Figure 12). It was an option where the samples worked in a liquid medium with abrasive elements. The high torque value was due to the hard abrasive particles. The use of a corrosive medium, i.e., sulfuric acid reduced the friction torque between the samples. For the normalized samples the values of the friction torque were similar to those of the C45 steel elements. For steel after hardening and high tempering, the friction values were analogous to the previous two cases. The friction torque for the samples after hardening and low tempering increased almost linearly with the successive considered cases, the values for samples where sulfuric acid was used were much higher than for distilled water as a medium. The results for nitrided elements were similar to those for steel 45, here also the highest values for mechanical and abrasive inputs were obtained. The values of the friction torque for chromium-plated samples were characterized by the highest values obtained among all. Such high values can be caused by a significant increase in the value of the Rv parameter.

### 3.3. Wear Test Results

**Figure 13:**
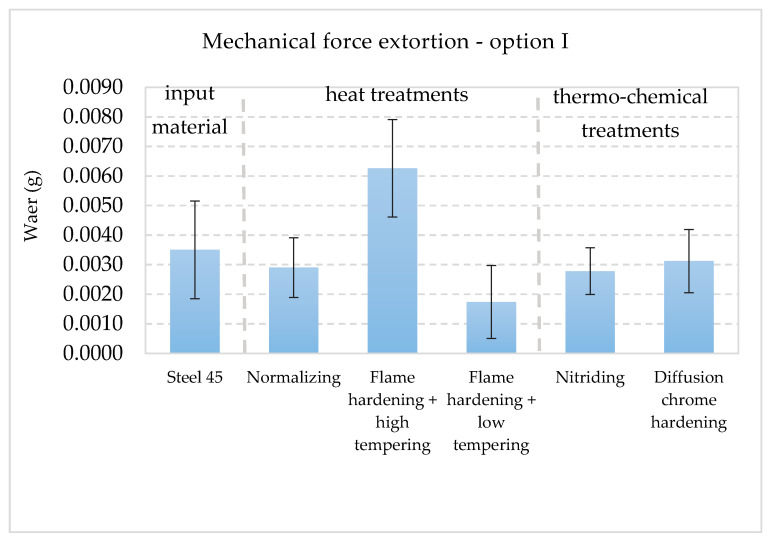
Wear I_M_ values in the conditions of mechanical extortion (no action from abrasive and corrosive agents) [40].

**Figure 14:**
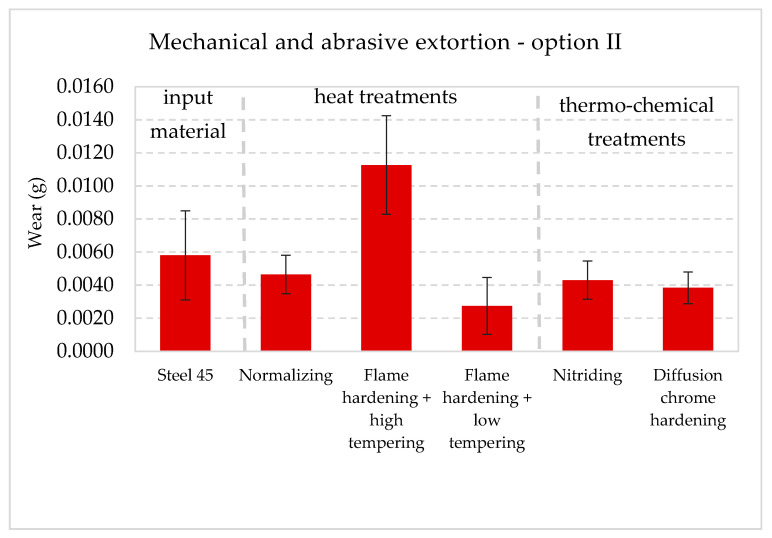
Wear I_M_ + I_s_ in the conditions in changeable mechanical and abrasive conditions (no action from corrosive agents) with 90% confidence semi-intervals [40].

**Figure 15:**
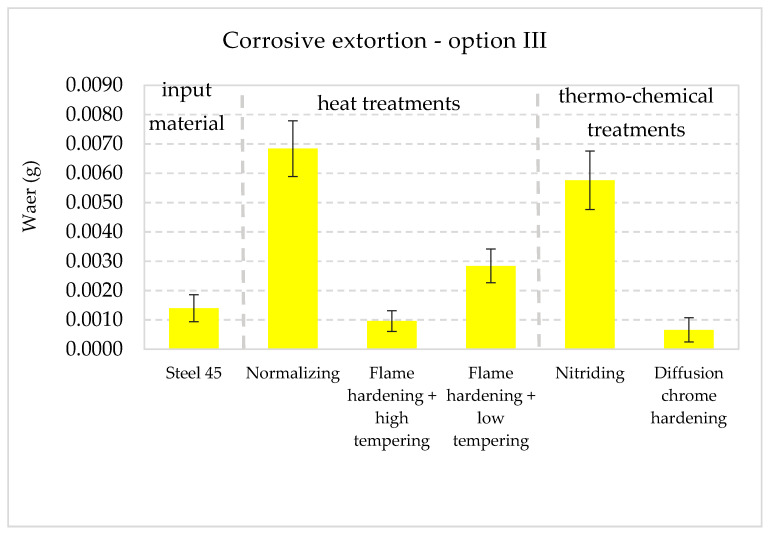
Wear I_C_ values in the conditions in corrosive extortion (no action from mechanical and abrasive agents) with 90% confidence semi-intervals [40].

**Figure 16:**
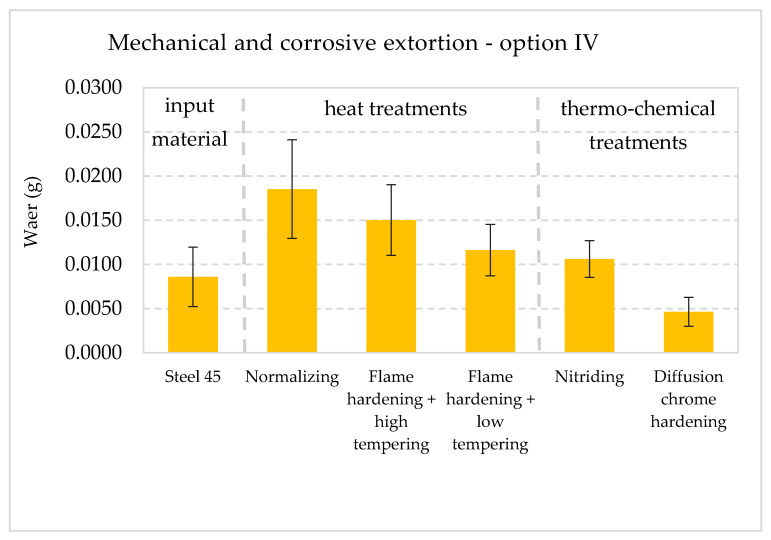
Wear I_M_ + I_C_ in the conditions in mechanical and corrosive conditions (no action from abrasive agents) with 90% confidence semi-intervals [40].

**Figure 17:**
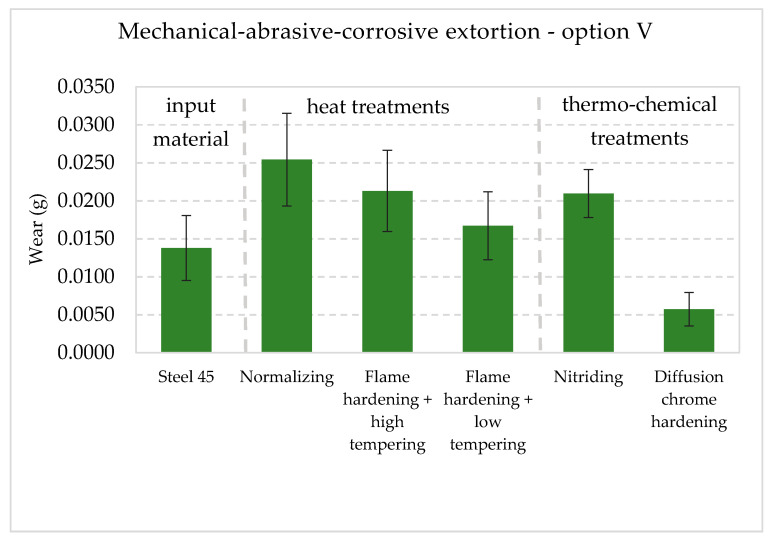
Wear I_M_ + I_C_ + I_A_ in the conditions in mechanical, abrasive and corrosive conditions with 90% confidence semi-intervals [40].

**Figure 18:**
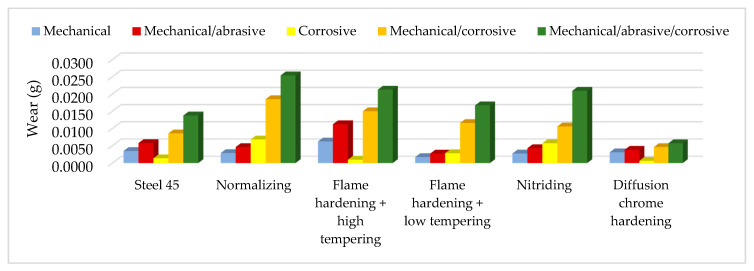
Collective presentation of wear results for all extortions [40].

The collective presentation of all values of wear for all analyzed types of treatment and all methodological options of the experiment is shown in Table 11. Figure 13, Figure 14, Figure 15, Figure 16, Figure 17 and Figure 18 present graphically all values of wear, separately for each experiment. The results were categorized into three groups in the following order: group I is the input material, i.e., the C45 steel, the second group is the options of heat treatments used, whereas group III contains results for heat and chemically treated samples.

The results of wear in mechanical extortion for specific options of treatment indicate their clear differentiation (Figure 13). The layer resulting from hardening and low tempering appeared to be the most resistant to wear in such conditions (the least wear). The values of wear for nitrided and normalized layers were at a similar level. The most worn layer was the one, which was hardened and high tempered. The wear of this layer appeared to be bigger than the result obtained for the input material, i.e., the C45 steel. The result pointed at the fact that after the above mentioned treatment, the intensity of mechanical wear had increased. The next experiment presents the implementation of the second methodological option. The wear resulted from mechanical and abrasive extortion. Figure 14 graphically presents the quantitative results.

Detailed analysis of data and figures displayed in the illustration led to the conclusion that simultaneous mechanical and abrasive extortion only in case of hardened and high tempered layer led to bigger wear than in the reference material, i.e., the C45 steel samples. The following was not difficult to notice, while comparing wear values in the surface layers in option I where the extortion was mechanical, with option II, where there was an additional abrasive factor:

(1) Radical increase in wear for option II,
(2) Values of wear in increasing order were similar to option I, except for the chrome hardened layer.

In the case of the chrome hardened layer and slightly less so in cases of the hardened and high tempered layers, the increase in wear was only moderate, caused by abrasion. The next experiment (research option III) included tests with corrosive agent only (no mechanical or abrasive extortion). The results are presented in Figure 15.

The results indicate a great differentiation of resistance to this kind of corrosive extortion of the surface layers of samples in different options of surface treatment. The smallest value of corrosive wear was noted in the chrome hardened layer. It is at least ten times less than the biggest recorded value, which was noted for the normalized layer. Very small values of wear were noted in hardened and high tempered layers, and the C45 steel (which had not undergone any treatment). Not only corrosive, but also nitrided layers had little resistance to corrosive forcing.

Experiment IV (Figure 16) indicates tests with the presence of both mechanical (force-kinetic) and corrosive agents but without the participation of the abrasive agent. The results obtained following the research indicate the presence of activities of mechanical extortion and the aggressive agent. The biggest wear was noted in the case of the normalized layer and the hardened and high tempered layer. Chromium layer proved to be the most resistant to wear in the analyzed conditions. The value of wear for this layer is twice as small as the wear of the surface layer in the C45 steel.

The last of the analyzed methodological options is the experiment in which all agents were present at the same time. Therefore, wear was the result of simultaneous action of mechanical, abrasive and corrosive extortion. The results are presented in Figure 17. The presented results clearly indicate that the noted values of wear were the biggest, compared to the other analyzed variants of treatment obtained in previous research options.

Collective graphic presentation is highlighted in Figure 18. It clearly indicates that the modification of the condition of the surface layer of the samples working in a model friction node significantly influenced the differentiation of wear results in the analyzed contexts of tribological extortion. Depending on the conditions applied, the layers had varied resistance to wear.

**Figure 19:**
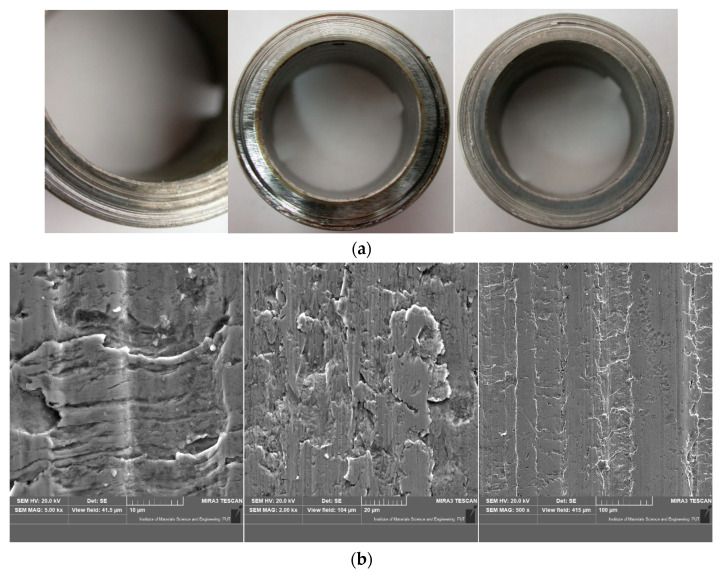
View of the wear surface of the samples for experiment VI; (**a**) view of the sample after test and (**b**) SEM images of the surface.

In the Figure 19 view of the sample and at various degrees of magnification of the surfaces after wear test (experiment VI) in different conditions are presented. For most of the sample dominant wear was caused by abrasive extortion.

Different types of the surface wear of individual samples were compared to the wear resistance in relation to the normalized sample for each extortion (Table 12). Since the C45 steel is dedicated to heat treatment and in the food industry is usually in the normalized state, values of wear resistance for the tested variants were referenced to the steel after the normalizing process. The increase in wear resistance for the flame hardening and low tempering layer under mechanical extortion is due to the high microhardness of this layer, the highest compressive stresses for all variants and the regular martensitic structure. High wear of the chrome layer consisting of iron-chromium carbide in relation to other surface layers, despite the highest hardness (among all tested machining variants) and characterized by high inherent stress, could be caused by high (over 2.5 times for height parameters) the increase in roughness parameters obtained after this treatment and the wear process taking place in a different way than in the case of other surface layers. After the mechanical wear process, this layer did not show any indentations or grooves caused by wear on its surface. Its surface became smooth and luminous (reflecting light), which would suggest that the surface was polished during this process for this layer. The low resistance of the flame hardened + high tempered layer to mechanical extortion could be caused by both the sorbitic structure (spherical cementite particles in the ferritic matrix), which is characterized by very high impact strength, an increase in yield strength and the lack of compressive stresses.

The greatest increase in resistance for mechanical/abrasive extortion was similar to the previous variant (mechanical wear) and obtained for the flame hardened and low-tempered layer. An additional abrasive factor in the form of hard sand grains made the layers characterized by high hardness (nitride and chrome-plated layer) in relation to the reference (normalized) layer showed a significant increase in resistance to this type of wear in relation to mechanical wear. This phenomenon may be explained by the fact that in softer layers (normalizing, steel 45, flame hardening and high tempering) hard abrasive particles caused microcutting of small volumes of metal of the surfaces tearing out its particles and plastic deformation and deformation of the surface layer.

The increase in resistance to corrosive wear in relation to the normalized layer for all tested variants of the surface layers were better than it. The highest increase (90%) was recorded for the chrome layer. This can be explained by the high ability of chromium to create a tight layer of passive oxides separating the metal surface from the corrosive environment, preventing the diffusion of corrosion products into the metal. Adamiak et al. [42] also obtained the best abrasive wear resistance for chromium cast iron, which was two times better than the surface layer cladded by the MMA ABRADUR 64 coated electrode and nine times better than HARDOX 400 steel.

Another layer that was highly resistant to corrosive wear (an increase of 85% compared to the normalized layer) is the flame hardened and highly tempered layer. In this case such a large increase in resistance in relation to the normalized layer may result from the lack of residual stress in this layer, which in corrosive environments may cause a significant increase in corrosion. The second factor determining high resistance may be the single-phase martensitic structure.

For the simultaneous occurrence of corrosive and mechanical effects the chrome layer had definitely the best anti-wear properties. The most important reasons for this phenomenon include the occurrence of low frictional resistance in relation to the resistance to mechanical excitation and high resistance to the corrosive agent. For the nitrided layer a 43% increase in resistance was noted in comparison to the reference material. This may indicate a poor-quality layer of the surface layer with nitrides. The thin layer of nitrides quickly deteriorated as a result of mechanical stress, exposing the layer directly below it to a simultaneous attack of corrosion and friction. Nevertheless, this layer in relation to the normalized layer, is characterized by a higher resistance to this type of wear.

The chrome layer had the best wear resistance for mechanical–corrosive–abrasive extortion. This is probably due to the considerable resistance of this layer to both mechanical and corrosive wear and the negligible influence of the abrasive agent on the increase in wear.

Wear caused by abrasion action connected with hard particles like sand is a major problem in many industrial applications, particularly in the areas of agriculture, mining, mineral processing, earth moving, etc. [43]. On the basis of the obtained results of particularly abrasive wear in laboratory conditions, the authors came to similar conclusions as [44] that larger parts are more effective in removing material. The view of the sample surface (Figure 19) after the research was similar to the view obtained by [20], which may indicate the presence of flake-like peeling and abrasive wear. Additionally for the C45 steel and second (II) extortion (mechanical and abrasive) research result in the form of wear (0.0058 g) was almost the same as in the case of the research results in [24]—wear around 0.0055 g. The abrasive wear process can be divided into two body and three body classes and into low and high stress systems with and without impact and it can work under the corrosion condition [1,4,5]. Many laboratory devices have been proposed to determine abrasion resistance of materials under simulated of field conditions in different types of tribological forcing factor like mechanical, abrasive and corrosive [42,45,46]. The authors checked the impact of both individual extortion on the wear of tested samples and the impact of two and three extortions. The main conclusion is that the wear effect caused by all factors—mechanical, abrasive and corrosive—is not a straight sum of values of wear.

Another factor influencing on the wear process is the condition of the surface layer, where the most significant impact has hardness, which is often associated with high abrasive wear resistance [47,48,49]. However it obviously is not only one phenomena resulting on wear resistance, so that it is not uncommon to find results in the literature where an increase in hardness may have no or even detrimental effects on abrasion resistance [50,51,52]. Additionally, surface roughness parameters measured before and after the test had changed. However, there is no correlation between the mass loss and these parameters [23].

## 4. Conclusions

Initial condition of the surface layer of mating elements of kinematic-motion nodes is a factor that seemed to be largely decisive about the intensity of the process of simultaneous tribological and corrosive wear. The noted changes in durability and reliability, not only of friction nodes and their elements, but also of the whole systems where they operated, i.e., machinery and equipment, were indirect effects of the process.

The tests indicate that when only mechanical extortion, or mechanical/abrasive extortion was present, the least losses were noted in the hardened and high tempered layer. The nitrided and chrome hardened layers proved to be well resistant, too. When it is only the corrosive agent that decides about the loss of mass, the strongest resistance was shown by the chrome hardened layer, and hardened and high tempered one. In cases of mechanical–corrosive and mechanical–abrasive–corrosive forcing options, the chrome hardened layer was decisively the most resistant.

The analysis proves also that the wear effect caused by joint mechanical, abrasive, corrosive and corrosive–abrasive extortion was not a straight sum of values of wear, but a sum of independent, separate forcing influences. Both the change in abrasive and corrosive extortion, and their joint action brought about a change in the intensity of use. It was also a confirmation of the presence of friction forces in complex processes (including friction–corrosive) and synergistic (strengthening) presences, which intensify the process of wear.

## Figures and Tables

**Table 1:** Selected works in the field of steel wear testing.

Type of Wear	Tested Material	Abrasive	References
abrasive	bainitic steel	-	[20]
abrasive, corrosive, erosive	steel 45	fused alumina	[21,22,23,24,25,26]
abrasive, corrosive		-	
abrasive, corrosive, erosive	alloy cast iron	fused alumina	[22,26]
abrasive, corrosive	stainless steel	Al_2_O_3_	[27,28,29]
abrasive, corrosive		abrasive strip	[30]
abrasive, corrosive		2% mixture of silica sand in water	[31]
abrasive	mild steel	silica sand abrasive particles	[32]

**Table 2:** Chemical composition of the C45 steel [1].

Content (%)								
C	Mn	Si	P	S	Cr	Ni	Mo	Cu
0.45–0.50	0.50–0.58	0.17–0.37	≤0.04	≤0.04	≤0.30	≤0.30	≤0.10	≤0.30

**Table 3:** Mechanical and physical properties of the C45 steel [1].

Resistance to Strain	Plasticity Limit	Elongation	Impact Resistance	Hardness	Density	Thermal Conduction Coefficient	Modulus of Elasticity
R_m_	R_e_	A	KU	HV	ρ	l	E
MPa	MPa	%	J	HV 10	g·cm^−3^	W·m^−1^·K^−1^	GPa
≥600	≥355	16	≥32	240	7.821	48.1	206

**Table 4:** The used options of heat and thermochemical treatment [40].

No.	Treatment Option	Parameters of Basic Types of Treatment			Additional Treatment
		Temp. T_o_ (°C)	Time t_o_ (h)	Cooling/Ambience	
1	Normalizing	840	1.2	in the air	-
2	Flame hardening + high tempering	880 580	7 min. 1	in water and in the air	-
3	Flame hardening + low tempering	880 170	7 min. 1.5	in water and in the air	-
4	Nitriding	530	5	atmosphere of ionized nitrogen	normalizing before nitriding; at the temperature of 840 °C, cooling in the air
5	Diffusion chrome hardening	1000–1050	4	chrome hardening powder	heat hardening after chrome hardening; hardening at 850 °C tampering at 160 °C (in oil) for 2 h

**Table 5:** Assumed values of input parameters [40].

No.	Parameter	Symbol	Value	Unit
1.	Pressure force	P	160	N
2.	Peripheral speed	v	0.2	m/s
3.	Corrosive liquid concentration	m	10%	H_2_SO_4_
4.	Abrasive material (sand) fraction	D	0.2–0.3	mm
5.	Stream of abrasive material inflow	Q	0.2	g/cm^2^·s
6.	Friction node temperature	*T*	23 ± 2	°C

**Table 6:** Methodological options of elementary tests [41].

Experiment	Extortion—The Factor			Model
	Mechanical	Abrasive	Corrosive	
I	+	−	−	I_I_ = I_M_
II	+	+	−	I_II_ = I_M_ + I_A_
III	−	−	+	I_III_ = I_C_
IV	+	−	+	I_IV_ = I_M_ + I_C_
V	+	+	+	I_V_ = I_M_ +I_C_ + I_A_

+ factor present in a given experiment; − factor absent from a given experiment; I_M_—mechanical extortion, I_A_—abrasive extortion, I_C_—corrosive extortion.

**Table 7:** HV01 hardness of the surface layer.

Condition of the Surface Layer	Mean Value	Standard Deviation	Confidence Half Interval	Coefficient of Variation
Steel 45	218.59	16.36	28.687	0.075
Normalizing	253.22	11.92	20.901	0.047
Flame hardening + high tempering	321.05	14.11	24.733	0.044
Flame hardening + low tempering	704.31	26.47	46.394	0.038
Nitriding	481.95	18.64	32.680	0.039
Diffusion chrome hardening	1918.75	75.78	132.834	0.039

**Table 8:** HV1 core hardness.

Condition of the Core	Mean Value	Standard Deviation	Confidence Half Interval	Coefficient of Variation
Steel 45	201.70	8.35	14.635	0.041
Normalizing	214.87	3.07	5.381	0.014
Flame hardening + high tempering	283.83	7.61	13.342	0.027
Flame hardening + low tempering	734.20	14.21	24.910	0.019
Nitriding	284.60	14.13	24.762	0.050
Diffusion chrome hardening	611.40	26.14	45.825	0.043

**Table 9:** The average results of surface roughness measurements before the wear test.

Parameter	Unit	Steel 45	Normalizing	Flame Hardening + High Tempering	Flame Hardening + Low Tempering	Nitriding	Diffusion Chrome Hardening
Ra	µm	0.567	0.670	0.683	0.667	0.730	1.423
Rtm	µm	3.147	3.780	3.810	3.743	4.757	7.997
Rz	µm	3.260	3.907	3.953	3.863	4.930	8.510
Rv	µm	4.093	4.877	4.753	4.563	7.243	10.833
Rq	µm	0.703	0.833	0.843	0.827	0.977	1.827
Rpk	µm	0.440	0.570	0.567	0.583	1.217	2.227
Rk	µm	1.750	2.153	2.187	2.233	2.217	4.400
Rvk	µm	0.900	1.023	1.007	0.900	1.093	1.697
Sa	µm	0.751	0.542	0.442	0.777	0.895	1.022
Sq	µm	0.910	0.687	0.550	0.950	1.145	1.320
Sp	µm	2.245	2.150	1.883	2.630	7.170	7.197
Sv	µm	2.340	2.040	1.803	3.160	3.680	4.900
St	µm	4.585	4.190	3.687	5.790	10.850	12.133
Sz	µm	4.410	4.027	3.603	5.305	9.435	10.960
Ssk	-	−0.233	−0.193	−0.231	−0.237	0.525	0.432
Sku	-	2.360	3.450	2.990	2.655	5.190	4.630

**Table 10:** Percentage changes in the Rv parameter depending on the tested variants of the surface layer.

Extortion	C45E Steel	Normalizing	Flame Hardening + High Tempering	Flame Hardening + Low Tempering	Nitriding	Diffusion Chrome Hardening
Mechanical	8%	−52%	276%	−17%	−58%	−69%
Mechanical/abrasive	21%	−38%	40%	60%	−50%	−62%
Corrosive	58%	69%	159%	64%	82%	48%
Mechanical/corrosive	92%	23%	7%	141%	−30%	−56%
Mechanical/abrasive/corrosive	56%	65%	162%	97%	−39%	−23%

**Table 11:** Presentation of test results of wear of samples in all analyzed options of the experiment g [40].

Type of Extortion	Mechanical	Mechanical/Abrasive	Corrosive	Mechanical/Corrosive	Mechanical/Abrasive/Corrosive
Research option	I	II	III	IV	V
C45 steel	0.0035	0.0058	0.0014	0.0086	0.0138
Normalization	0.0029	0.0046	0.0068	0.0185	0.0254
Hardening + high tempering	0.0063	0.0113	0.0010	0.0150	0.0213
Hardening + low tempering	0.0017	0.0027	0.0028	0.0116	0.0167
Nitriding	0.0028	0.0043	0.0058	0.0106	0.0210
Chrome hardening	0.0031	0.0038	0.0007	0.0046	0.0057

**Table 12:** Changes in wear resistance in % of various variants of treatments in relation to the normalized layer for individual extortion.

Extortion/Surface Layer	Steel 45	Normalizing	Flame Hardening + High Tempering	Flame Hardening + Low Tempering	Nitriding	Diffusion Chrome Hardening
Mechanical	−21	0	−117	41	3	−7
Mechanical/Abrasive	−26	0	−146	53	7	17
Corrosive	79	0	85	59	15	90
Mechanical/Corrosive	54	0	19	37	43	75
Mechanical/Abrasive/Corrosive	46	0	16	34	17	78
